# Children's Hospital, Nottingham

**Published:** 1899-02-04

**Authors:** 


### CHILDREN'S HOSPITAL, NOTTINGHAM.

In publishing the following letters, we would repeat what we have said before—that we make no charge and bring no complaint against the Children's Hospital at Nottingham. What we have done is this: We have stated that "we are strongly convinced of the desirability of all charges brought against hospitals being publicly met and openly investigated"; we have published a complaint which had been made against the Nottingham Children's Hospital, and which had been met in such a way as to leave the complainant unsatisfied, a thing which is always most injurious to any hospital; and in publishing this complaint we have picked out certain points to show that "the charge here made is far too serious to be passed over without full explanation." What we said was not said by way of endorsing the charge, but of showing that it was of sufficient gravity to demand a full and public explanation, and we are still of this opinion. We do not wish to bandy words, but we can hardly think that the public will be quite satisfied with the chairman's letter which we now publish. As to the case having been an "unimportant" one, that we leave the officials who signed the statement to settle with the Nottingham public. Of course, much depends upon the meaning of a word, but we would point out that unless the statements made by Miss Morrish as to the condition of the child are denied, which we do not gather to be the case, the word "unimportant" must have been used in a somewhat peculiar sense. Is it a fact that the child was suffering from erysipelas; that the disease was so severe as to cause swelling of the lips; that for days it was totally blind; that the erysipelas not only affected the head and face, but extended on to the chest, arms, and abdomen; that for its accommodation the isolation block was brought into occupation; and that the little patient was given the entire service of a probationer? If these things are true, then it may well be asked both by the subscribers and the friends of the patients what is meant by the word "unimportant"? We thank the chairman for his courtesy in suggesting that we should visit the hospital, and for his offer of additional information. But that is not the point. We are not the complainants, and all we urge is that, unless the charges made can be absolutely denied, a public inquiry should be made into the management of an institution in which such things can take place. As for the innuendo in the following letter, which suggests that the complaint does not come from Miss Morrish at all, we can only say that if the chairman has any reason to suspect bad faith, that is all the greater reason why any inquiry or any explanation should be given full publicity.

Morrish v. Children's Hospital, Nottingham.

The Chairman of the Board of Management writes: The writer of the article in The Hospital of January 21st on the above subject makes some deductions which are not, in our opinion, warranted by the facts of the case, and at the same time he has failed to do justice to the information supplied by this committee to your paper. He infers that because the child was baptized and from certain statements purporting to come from Miss Morrish the case was of a severe nature. May we ask him to read again the replies given to the Editor's questions, which bear not only the signatures of the chairman of this committee and its lady superintendent, but also those of the medical officers of the charity. These gentlemen, who may surely be deemed to be the best judges of the child's condition, state that the case was "unimportant." As against this, the account which, we repeat, purports to come from Miss Morrish, is accepted as a basis for his reasoning, although the chairman of this committee states in his letter that her (?) account contains "several inaccuracies introduced by inadvertence," &c. The fact of the child having been baptized soon after its admission was, in absence of explanation, calculated to mislead, and this it appears to have done in the case of the writer of the article we refer to. The child was baptized simply because the father regretted that the ceremony had been omitted in this child's case before it left home, and, as he expressed it, "no child of his had ever done this before." No suggestion was made as to baptism by any official, as the special need for it (such as early death) was not looked for. Miss Morrish was not responsible for the case, but she was selected by the lady superintendent to act under the sister in charge, with a view to test her capacity and fitness for a place on the staff. This is our view of a "sitter," though we recognise the fact that the term is somewhat ancient. The phrase "sole charge" was adopted from the Editor's letter bearing date December 22nd. Miss Morrish is wrong if she really said she had never received any instructions before she gave an enema, as she had been thoroughly taught how to do so in the hospital wards, and equally inaccurate if she also stated that she was not supervised when giving these in "isolation," neither should she have ignored the teaching she received in the use of the thermometer. Miss Morrish left the hospital, having, some days before her father's recall, been told that she could not be accepted to act on the staff. This lady, who is said to have written the account you publish and we received, asked very earnestly to be allowed at least to stay to complete the case. To add to the former inaccuracies named by us, we must assert that Miss Morrish was wrong if she stated she was not visited at night, or with the long intervals described by her. The writer also takes exception to the answer to Question 4. The answer to this was couched in the language used by us with the view of conveying what the custom of the hospital was so far as the isolation block was concerned. To this we would now add the fact that the sister in charge always sleeps in the isolation block when the case is serious. That this committee is fully alive to certain defects will be evident if the letter (October 28th) is read patiently. No mention is made in the article or elsewhere of the receipt of a letter (dated December 30th) by the Editor which travelled with the "Answers," and was written by the secretary. In this letter we ask the Editor or a representative to sift the matter, and offer additional information and solicit a visit to the hospital. We think that this letter should have been published with the other correspondence. This offer is still open. If the writer of the article still thinks he has "not got to the bottom of the matter" let him at least make use of this. We may add in conclusion that the committee, having very carefully investigated all the circumstances of the case, are of opinion that the lady superintendent was justified in placing Miss Morrish on isolation duty.

---

Miss L. M. Newill, matron of the Jenny Lind Infirmary, Norwich, writes: Seeing the view you took of the statement made by Miss Morrish (a probationer of three weeks' standing) in last week's Hospital, my first thought was to write a detailed letter giving my own experience of the Nottingham Children's Hospital; but knowing as I do the high standard of nursing which has always been maintained in the hospital, I felt such an answer would be out of place. Having known the hospital for eleven years, during six of which I had the privilege of working there under the past and present *régime*, I feel no words of praise could adequately express the excellent way in which the children are cared for, body and soul, and the great attention that is paid to the training of probationers even in the minutest details. Nottingham Children's Hospital is known far and wide, therefore I feel that such a statement, made as it was by a probationer during her trial month, can in no way injure its high reputation. In writing this I am convinced I am only expressing the sentiments of all those who have worked for any length of time in the hospital.

